# Whole-Exome Analysis Identifies Candidate Genes Associated With Diabetic Retinopathy

**DOI:** 10.1167/iovs.67.8.25

**Published:** 2026-07-08

**Authors:** Ning Li, Long Liu, Wen Sun, Di Liu, Haibin Li, Changwei Li, Xiao Wang, Jianguang Ji, Yalu Wen, Deqiang Zheng

**Affiliations:** 1 Department of Epidemiology and Health Statistics, School of Public Health, Capital Medical University, Beijing, China; 2 Department of Statistics, University of Auckland, Auckland, New Zealand; 3 Beijing Key Laboratory of environment and aging, Capital Medical University, Beijing, China; 4 Department of Health Statistics, School of Public Health, Binzhou Medical University, Yantai, Shandong, China; 5 Department of Public Health and Medicinal Administration, Faculty of Health Sciences, University of Macau, Taipa, Macau, China; 6 Medical Research Center, Beijing Chaoyang Hospital, Capital Medical University, Beijing, China; 7 Department of Epidemiology, O'Donnell School of Public Health, University of Texas Southwestern Medical Center, Dallas, Texas, United States; 8 Center for Primary Health Care Research, Lund University, Region Skåne, Malmö, Sweden

**Keywords:** diabetic retinopathy, *FRZB*, multiomics, whole exome sequence

## Abstract

**Purpose:**

Despite adequate glycemic control, a proportion of patients develop diabetic retinopathy (DR), suggesting the contribution of other mechanisms. To explore genetic associations with DR, we conducted a whole-exome sequencing (WES) study of DR using a dual-control design in individuals of European ancestry.

**Methods:**

Leveraging UK Biobank WES data, we implemented a dual-control design to identify candidate genes associated with DR, comparing cases with both diabetes controls and the general population. Rare variant associations were tested at gene-level using SAIGE-GENE+, and common variants were analyzed via PLINK2. Significant findings were assessed through the exclusion of self-reported cases, leave-one-variant-out (LOVO) analysis, time-to-event analysis, transcriptomics analysis, virtual knockout experiments, proteomics, and protein–protein interaction (PPI) analysis.

**Results:**

Exome-wide gene-based association analysis identified *FRZB* (frizzled-related protein) as a candidate gene potentially associated with DR in comparisons with diabetic controls (odds ratio [OR] = 1.09; 95% confidence interval [CI], 1.04–1.15; *P* = 2.55 × 10^−7^). The observed association showed generally consistent patterns across several sensitivity analyses, including exclusion of self-reported cases, LOVO analyses, and Cox proportional hazards models. In addition, transcriptomic, proteomic, virtual knockout, and PPI analyses provided complementary exploratory evidence supporting a possible involvement of *FRZB* in DR-related biological processes. Additionally, single-variant analysis revealed two loci, *FAM160A1* (4:151662612:C:G) and *HK1* (10:69300854:A:G), associated with DR in the general population (OR = 1.82 and OR = 1.50, respectively). However, multiomics support for these signals was limited.

**Conclusions:**

This multilayered genetic study identified *FRZB* as a candidate gene potentially associated with DR. However, this finding should be interpreted cautiously, and further experimental studies and independent replication are needed to clarify the potential relevance of *FRZB* to DR.

Diabetic retinopathy (DR) remains a leading cause of blindness among working-age adults worldwide.^1^ The global prevalence is projected to rise from 103 million cases in 2020 to approximately 160 million by 2045.^2^ Tight glycemic control is regarded as the most effective strategy for preventing DR; however, despite strict blood glucose regulation, some individuals still develop DR,^3^ emphasizing the need to identify novel factors of DR.

Beyond traditional metabolic factors, genetic predisposition plays a critical role in the development of DR. Numerous genome-wide association studies (GWASs) have identified multiple loci associated with DR, including *VEGFA*,^4^ *ICAM1*,^5^ and *AKR1B1.*^6^ However, most GWAS signals involve common variants located in non-coding regions, limiting direct functional interpretation. In addition, GWASs are generally underpowered to detect rare variants associated with DR. Whole-exome sequencing (WES) offers complementary advantages by targeting protein-coding regions, enabling the identification of rare variants and coding common variants with clearer potential functional relevance. Nevertheless, prior WES studies of DR (*n* = 70–107)^7^^,^^8^ have been constrained by small sample sizes, limiting statistical power.

In this context, we leveraged large-scale WES data to systematically evaluate both rare and common coding variants using a dual-control framework, comparing DR cases against both (1) diabetes-specific controls (individuals with diabetes but without DR) and (2) general population controls (individuals without diabetes and DR). This framework specifically identifies DR-associated genetic signals after accounting for glycemic status.

## Materials and Methods

### Study Design

**Figure 1.**
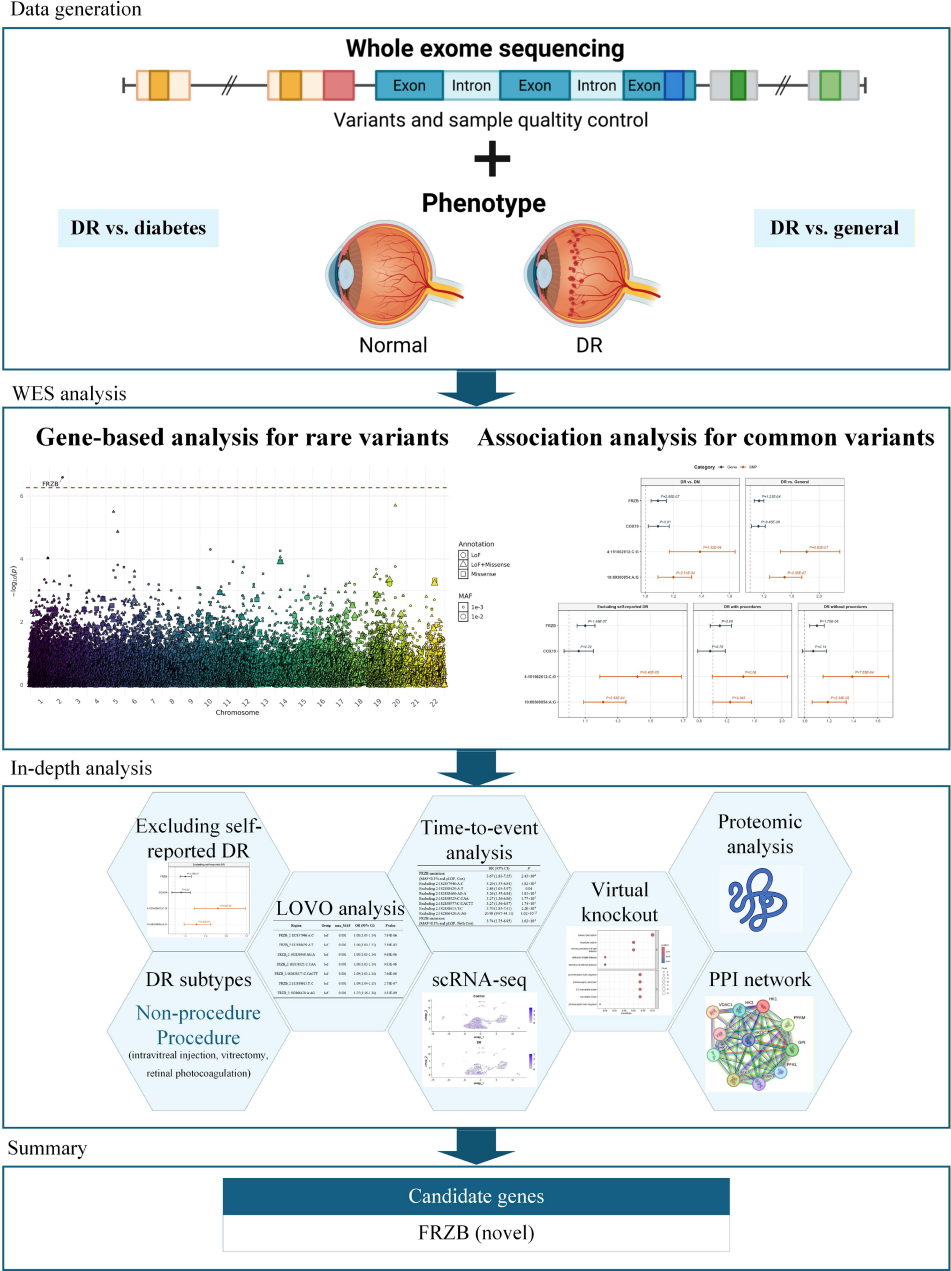
Study design.

We implemented a multistage analytical framework to identify candidate genes associated with DR using WES data from the UK Biobank. First, we adopted a dual-control strategy by comparing DR cases against both the general population and individuals with diabetes, aiming to evaluate genetic effects associated with DR while accounting for glycemic status. To enhance phenotype accuracy, we repeated analyses after excluding DR cases identified solely through self-report. Next, DR cases were stratified based on ophthalmic procedure recodes, including vitrectomy, retinal photocoagulation, and intravitreal injection. These procedure-defined subgroups were used as exploratory proxies for treatment-requiring DR, rather than formal disease staging, to assess potential heterogeneity in genetic effects. We then performed leave-one-variant-out (LOVO) analyses to assess whether observed gene-level associations were driven by single variants or cumulative effects. To validate the robustness of these findings, we conducted time-to-event analyses and transcriptomic and proteomics analyses. Virtual knockout experiments of identified genes were performed to identify downstream transcriptional changes and enriched biological pathways. Finally, we investigated whether genes within protein–protein interaction (PPI) networks of candidate targets were also associated with DR. This study was conducted using data from the UK Biobank under approved application number 95259. The overall study design is illustrated in Figure 1.

### Study Population

The UK Biobank is a prospective cohort study that recruited 502,131 participants between 2006 and 2010 from 22 assessment centers across the United Kingdom. WES was processed by the Regeneron Genetics Center. In brief, DNA was extracted from blood samples and stored in an automated sample biobank at −80°C. The samples were sequenced using 75-base-pair (bp) paired-end reads with two 10-bp index reads on a NovaSeq 6000 system (Illumina, San Diego, CA, USA).^9^

A total of 53,477 individuals were identified as having diabetes, based on self-reported medical history, the administration of anti-diabetic drugs, blood glucose and HbA1c levels, and hospital inpatient records (Supplementary Table S1). We defined two distinct control groups for downstream analyses. For the diabetes control group, individuals with gestational diabetes or lacking a recorded date of diabetes onset were excluded. In addition, participants with diabetic microvascular complications (retinopathy, nephropathy, or neuropathy) prior to diabetes diagnosis were removed. After these exclusions, 49,609 participants remained eligible, including 5096 individuals identified with DR and 44,513 diabetes controls without DR. For the general population control group, individuals without a diagnosis of diabetes (*n* = 448,654) were selected as controls (Supplementary Fig. S1). The diagnosis of DR was based on self-report; International Classification of Diseases, 10th Revision (ICD‑10) codes; and ICD‑9 codes (Supplementary Table S1).

### WES Quality Control

The WES data underwent rigorous quality control (QC) at both variant and sample levels. For variant QC, we retained variants for which ≥90% of genotypes had a read depth of 10 or greater and maintained a minimum call rate of 90%, while excluding variants showing significant deviation from Hardy–Weinberg equilibrium (*P* < 1 × 10^−15^). These filters yielded 20,716,751 high-quality variants for analysis. At the sample level, we excluded individuals with >10% missing genotype rates and restricted the analysis to participants of genetically inferred European ancestry after removing individuals with sex discordance between self-reported and genetically inference. After excluding related individuals (kinship coefficient ≥ 0.0884, corresponding to second-degree relatives or closer),^10^ 366,235 individuals remained with high-quality WES data. Following these filters and after merging phenotype and genetic data, the final analysis included 33,624 participants in the diabetes control group and 333,200 participants in the general population control group (Supplementary Fig. S1).

### Variant Annotation

Rare variants with minor allele frequency (MAF) <0.01 were functionally annotated using SnpEff,^11^ retaining the most severe predicted consequence for each gene transcript. Variants classified as stop gained, stop lost, start lost, splice donor, splice acceptor, or frameshift were grouped as predicted loss of function (pLoF). Missense variants were considered likely deleterious if consistently predicted as damaging by multiple tools, including SIFT,^12^ PolyPhen2 HDIV and PolyPhen2 HVAR,^13^ LRT,^14^ and MutationTaster.^15^ For single-variant association testing, common variants (MAF ≥ 0.01) annotated as “exonic” or “exonic;splicing” by the ANNOVAR tool were included.^16^

### Exome-Wide Gene-Based Analysis for Rare Variants

We performed an exome-wide gene-based analysis for rare variants to identify genes influencing DR independently of glycemic control. Using SAIGE-GENE+,^17^ we conducted gene-based tests incorporating six distinct variants masks combining three functional annotation (pLoF alone, missense alone and pLoF+missense) and two MAF thresholds (0.1% and 1%). To account for population structure and relatedness, we constructed a sparse genetic relationship matrix (GRM) from the linkage disequilibrium (LD)-pruned hard-called variants (relatedness cutoff = 0.05), which was used to fit the null logistic mixed model for variance ratio estimation, with adjustments for sex, age, HbA1c, and the first 10 principal components (PCs). Association testing for each gene was evaluated using SKAT,^18^ burden,^19^ and SKAT-O^20^ models, with SKAT-O results reported as primary findings due to its adaptive balance between burden and variance-component tests. We set the false discovery rate (FDR)-corrected significance level at 0.05.

To establish glycemic independence, we compared their association *P* values across both control groups to identify the genetic effects accounting for glycemic status. Second, we directly examined the associations between significant genes and HbA1c levels at the baseline assessment using the WES data. FDR correction was applied at the gene level based on the set of genes identified in the discovery stage.

### Single Association Analysis for Common Variants

We conducted single-variant association testing for common variants annotated as “exonic” or “exonic;splicing” using a logistic regression model implemented in PLINK2.^21^ Variants with FDR-adjusted *P* < 0.05 were considered statistically significant. We applied the same approach used for rare variants to identify significant common variants: (1) comparative analysis across both control groups (diabetes vs. general population), and (2) examination of HbA1c associations. Subsequently, we performed LD clumping on significant variants using an *r*^2^ threshold of <0.1 within 500-kb windows. Finally, we mapped the lead variants to genes using ANNOVAR.^22^

### DR Refinement and Exploratory Subtype Analyses

To ensure the robustness of our findings, we excluded DR cases identified solely based on self-reported information and repeated the analyses to minimize potential misclassification bias. In addition, using procedure codes from the Office of Population Censuses and Surveys Classification of Interventions and Procedures (OPCS-4) within the UK Biobank, we identified DR patients with records of ophthalmic interventions, including intravitreal injection, vitrectomy, and retinal photocoagulation (Supplementary Table S2). Because standardized ophthalmic grading data for DR severity were unavailable in the UK Biobank, these procedure records were not interpreted as definitive markers of DR stage but rather as potential indicators of treatment-requiring disease manifestations. DR cases were then grouped based on the presence or absence of these procedures, and analyses were performed separately as exploratory assessments of potential heterogeneity in genetic associations across procedure-defined groups.

### Leave-One-Out Analysis

To determine whether gene-based associations with DR were driven by a single variant or reflected the cumulative effect of multiple variants, we conducted LOVO analyses. For each gene-based mask comprised of *n* variants, one variant was sequentially excluded to create *n* alternative masks, each omitting a different variant. Association tests were then repeated using each reduced mask to assess the influence of single variants on the overall gene–DR association.

### External Support Analyses in FinnGen

To explore the consistency of our findings, we used summary-level data from the FinnGen consortium (v12) for the phenotype “Diabetic retinopathy (more control exclusions),” which included 14,142 DR cases and 59,084 diabetes controls. Details of quality control procedures are described in previous publications.^23^ DR was identified using ICD-10 H36.0 and ICD-9 3620. Controls were defined as individuals with diabetes but without a diagnosis of diabetic complications. We leveraged the FinnGen GWAS summary statistics to evaluate association signals within the genomic region of each candidate gene. Specifically, all SNPs located within the gene boundary were examined. A gene was considered replicated if at least one SNP in its genomic region showed the evidence of association with DR (*P* < 0.05/the number of genes).^24^^,^^25^

### Cox Proportional Hazard Analysis for DR

To evaluate the longitudinal association between gene mutations and the risk of developing DR, we conducted Cox proportional hazards models among UK Biobank participants with diabetes. The exposure was defined as the burden of rare variants in the candidate genes or common variants, and the outcome was incident DR. Follow-up time was calculated from the date of diabetes diagnosis to the first occurrence of DR diagnosis, death, lost, or the end of follow-up, whichever came first. Hazard ratios (HRs) and 95% confidence intervals (CIs) were estimated using Cox proportional hazards models adjusted for age, sex, and HbA1c. The LOVO analyses were conducted by iteratively excluding individual variants to assess whether gene-level associations were driven by single variants or by aggregated effects of multiple variants. In addition, given the low frequency of variants and potential sparse event bias, we additionally applied a bias-reduced penalized likelihood approach using the Firth correction in Cox regression to address potential small sample bias and sparse event issues.

### Single-Cell Transcriptome Analysis and Virtual Knockout Analysis

We obtained single-cell RNA sequencing (scRNA-seq) data from the retinal tissue of *Macaca fascicularis* from the Gene Expression Omnibus (GEO) database (GSE168908).^26^ Standard quality control, normalization, integration, and clustering procedures were carried out using the Seurat package^27^ in R (R Foundation for Statistical Computing, Vienna, Austria). Cells were filtered according to three criteria: (1) 200 to 3000 detected genes per cell (nFeature_RNA), (2) total unique molecular identifier (UMI) counts (nCount_RNA) between 300 and 20,000, and (3) mitochondrial gene content below 30%. Following normalization and integration, principal component analysis (PCA) was performed for dimensionality reduction. Cell types were annotated manually based on canonical marker genes as reported in the original study.^26^ To identify cell-type–specific transcriptional differences between samples of diabetes and without diabetes, we used the Seurat FindMarkers function. Genes were considered differentially expressed if they had an adjusted *P* < 0.05 and an absolute average log fold change (|avg_logFC|) ≥ 0.5.

To investigate the functional consequences of candidate gene perturbation, we conducted virtual knockout analyses using the R package scTenifoldKnk,^28^ a machine learning framework based on scRNA-seq data. Cell types showing differential expression of the candidate genes were first selected for downstream analysis. For each selected cell type, the top 2000 highly variable genes were used to construct gene regulatory networks (GRNs). The candidate gene was then virtually knocked out, and differences between the perturbed and original GRNs were quantified through manifold alignment to identify significantly affected genes (*P*_adj_ < 0.05).^29^ Subsequently, functional enrichment analyses were performed using the R package clusterProfiler (with 2000 highly variable genes as background) to identify enriched pathways among differentially affected genes and thereby evaluate downstream functional alterations linked to candidate gene perturbation.

### Proteomics Analysis and PPI Networks

We leveraged proteomics data from the UK Biobank to investigate the association between circulating protein levels and the risk of developing DR. Participants diagnosed with type 2 diabetes who had available proteomic measurements were included in the analysis. A Cox proportional hazards model, adjusted for age at baseline, sex, HbA1c, and duration of diabetes, was applied to assess the effect of protein expression levels on incident DR. Protein expression levels were treated as continuous variables, and HRs were estimated to quantify their association with DR risk. To minimize potential reverse causation, we performed a sensitivity analysis excluding participants who developed DR within the first year of follow-up. In addition, to further account for cardiometabolic comorbidities, we conducted a multivariable model additionally adjusting for hypertension status, total cholesterol, and vascular or heart problems.

To provide complementary biological validation, we conducted PPI network analyses. For each identified candidate gene, interacting proteins were retrieved from the STRING database (species: *Homo sapiens*; minimum interaction score = 0.4). We then evaluated whether these interacting proteins had prior evidence of association with DR by querying the Open Targets Platform (https://platform.opentargets.org/) and DisGeNET (https://disgenet.com/), thereby providing indirect biological evidence supporting the relevance of the candidate targets. Furthermore, we performed enrichment analyses to characterize biological pathways jointly enriched by the candidate targets and their interacting proteins with prior DR-related evidence, aiming to elucidate the functional contexts through which these genes may contribute to disease pathogenesis. Finally, as in previous articles,^30^^,^^31^ we used the Human Protein Atlas (https://www.proteinatlas.org/) and GeneCards (https://www.genecards.org/) to examine whether a candidate gene is enriched in eye or retinal tissues, thereby strengthening the possibility of its role in DR.

## Results

After quality control, a total of 3320 individuals with DR were identified. Among these cases, 62.2% were male, with a mean ± SD age of 60.5 ± 6.74 years. In the diabetes control analysis, the control group was comprised of 30,304 individuals with diabetes but without DR, of whom 60.6% were male and the mean age was 59.5 ± 7.11 years. In the general population control analysis, the control group included 329,880 individuals without DR (44.8% male) and with a mean age of 56.7 ± 7.97 years. WES examined 18,233 protein-coding genes in the diabetes control group and 19,038 in the general population control group.

### Exome-Wide Gene-Based Analysis for Rare Variants

**Figure 2.**
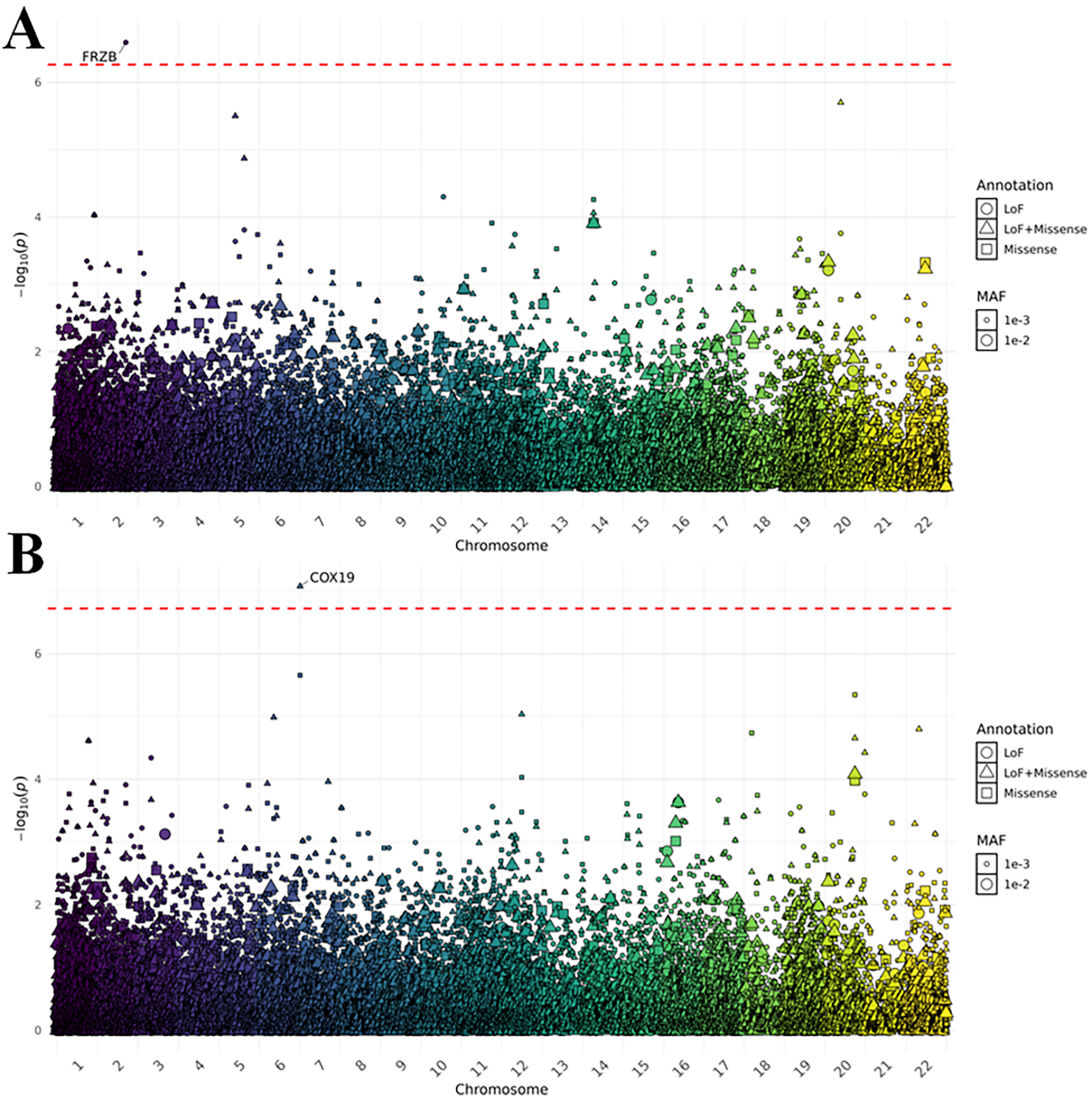
Manhattan plots. (**A**) The results of gene-based analysis for DR (DR vs. diabetes). (**B**) The results of gene-based analysis for DR (DR vs. general population).

**Figure 3.**
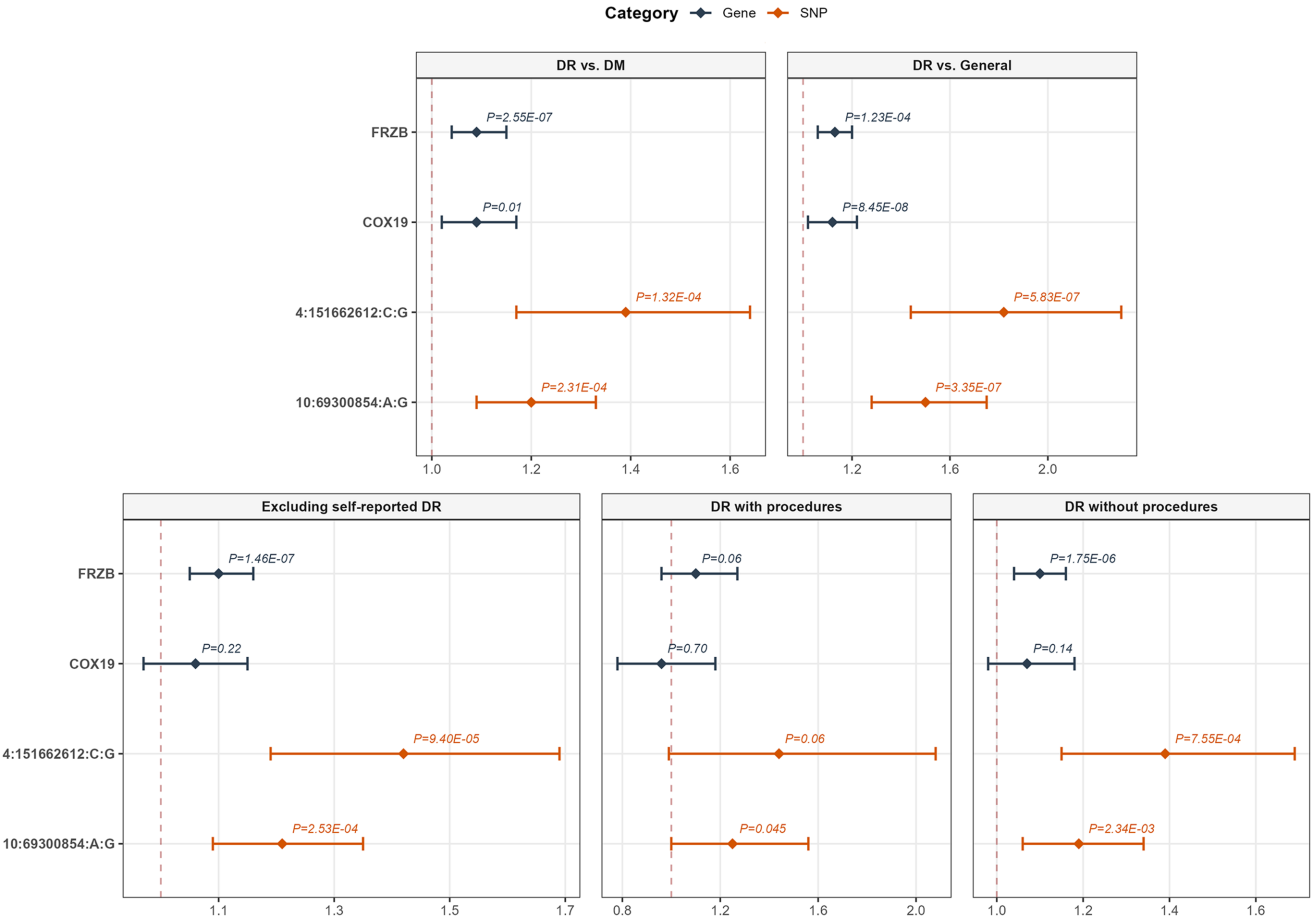
Genetic associations of candidate genes and variants with DR across multiple analytical strata. The *top panels* display associations relative to individuals with diabetes and the general population. The *bottom panels* show results after excluding self-reported DR cases and stratifying by the presence or absence of ophthalmic procedures.

The gene-based rare variant analysis revealed distinct genetic associations when using different control groups. Compared to diabetes controls, pLoF mutations in *FRZB* (frizzled-related protein) showed a significant association with DR (MAF < 0.1%; OR = 1.09; 95% CI; 1.04–1.15; *P* = 2.55 × 10^−7^; FDR = 0.01). In contrast, using the general population as controls identified *COX19* as significantly associated with DR (missense + pLoF, MAF < 0.1%; OR = 1.12; 95% CI, 1.02–1.22; *P* = 8.45 × 10^−8^; FDR = 0.005) (Fig. 2). Comparative analysis of the raw *P* values across both control groups suggested that pLoF mutations in *FRZB* showed associations with DR (*P*_diabetes_ = 2.55 × 10^−7^ vs. *P*_general_ = 1.23 × 10^−4^) (Fig. 3, Supplementary Table S3). By contrast, the association for *COX19* was markedly reduced when restricting controls to individuals with diabetes (*P* = 0.01) (Fig. 3, Supplementary Table S3). Further analyses indicated no association with HbA1c levels for *FRZB* (pLoF variants, *P* = 0.63) or for *COX19* (missense + pLoF variants, *P* = 0.81) was associated with HbA1c levels (Supplementary Table S3), suggesting that the observed genetic associations may not be mediated by glycemic status, although this interpretation is limited by the use of a single HbA1c measurement.

### Single Association Analysis for Common Variants

We utilized PLINK2 to assess the association between common variants and DR across different control groups. When individuals with diabetes were used as the control group, no genome-wide significant variants were identified after FDR correction. In contrast, with the general population as controls, two variants reached the significance threshold: 4:151662612:C:G (OR = 1.82; 95% CI: 1.44–2.30; *P* = 5.83 × 10^−7^; FDR = 0.03) and 10:69300854:A:G (OR = 1.50, 95% CI, 1.28–1.75; *P* = 3.35 × 10^−7^; FDR = 0.03), mapping to *FAM160A1* and *HK1* (hexokinase 1), respectively (Fig. 3, Supplementary Table S3). When the analysis was restricted to individuals with diabetes as controls, the associations for both variants were attenuated but remained statistically significant: 4:151662612:C:G (OR = 1.39; 95% CI, 1.17–1.64; *P* = 1.32 × 10^−4^); 10:69300854:A:G (OR = 1.20; 95% CI, 1.09–1.33; *P* = 2.31 × 10^−4^) (Fig. 3, Supplementary Table S3). We further evaluated the associations between these variants and HbA1c. Variant 10:69300854:A:G showed a strong association with HbA1c (*P* = 3.47 × 10^−150^) (Supplementary Table S3). Although the variant remained significantly associated with DR after adjustment for HbA1c, these findings should be interpreted cautiously, as the single HbA1c measurement may not fully capture long-term glycemic exposure.

In the UK Biobank, only two HbA1c measurements were available, and the second measurement was missing in the majority of participants (∼97%), precluding further adjustment analyses. Among individuals with both measurements available, the two HbA1c values showed a significant correlation (Spearman *r* = 0.64), suggesting some longitudinal consistency of glycemic status over time. Nevertheless, residual confounding related to long-term glycemic exposure cannot be excluded.

### DR Refinement and Exploratory Subtype Analyses

After excluding 394 DR cases identified solely through self-report, we repeated the analyses using individuals with diabetes as controls. In the gene-based analysis, *FRZB* remained significantly associated with DR (OR = 1.10; 95% CI, 1.05–1.16; *P* = 1.46 × 10^−7^), whereas the association for *COX19* was no longer significant (OR = 1.06; 95% CI, 0.97–1.15; *P* = 0.22). Single-variant analyses showed that both 4:151662612:C:G (OR = 1.42; 95% CI, 1.19–1.69; *P* = 9.40 × 10^−5^) and 10:69300854:A:G (OR = 1.21; 95% CI, 1.09–1.35; *P* = 2.53 × 10^−4^) remained significantly associated with DR (Fig. 3).

We further performed exploratory analyses stratifying DR cases based on ophthalmic procedures. Among DR cases, 2323 patients had no records of these procedures, whereas 603 had received ophthalmic intervention. In comparisons with diabetes controls, pLoF variants in *FRZB* were significantly associated with DR in the non-procedure group (OR = 1.10; 95% CI, 1.04–1.16; *P* = 1.75 × 10^−6^), but not in the procedure group (OR = 1.10; 95% CI, 0.96–1.27; *P* = 0.06), although the direction of effect remained consistent. *COX19* was not significantly associated with DR in either subgroup: non-procedure (OR = 1.07; 95% CI, 0.98–1.18; *P* = 0.14); procedure (OR = 0.96; 95% CI, 0.78–1.18; *P* = 0.70). For single-variant analyses, 4:151662612:C:G remained significantly associated with DR in the non-procedure group (OR = 1.39; 95% CI, 1.15–1.69; *P* = 7.55 × 10^−4^), but not in the procedure group (OR = 1.44; 95% CI, 0.99–2.08; *P* = 0.06). In contrast, 10:69300854:A:G showed nominal associations in both subgroups: non-procedure (OR = 1.19; 95% CI, 1.06–1.34; *P* = 0.002); procedure (OR = 1.25; 95% CI, 1.00–1.56; *P* = 0.045) (Fig. 3).

### Leave-One-Out Analysis

Given that the results for COX19 were not significant after the exclusion of self-reported cases, we only conducted LOVO analysis for pLoF variants in *FRZB* among individuals with diabetes, adjusting for age, sex, HbA1c, and the first 10 principal components. The associations with DR remained significant regardless of which individual variant was excluded (Supplementary Table S4).

### External Support Analyses in FinnGen

To explore whether similar association patterns could be observed in an independent dataset, we queried GWAS summary statistics from the FinnGen consortium. The variants rs909949311 (in *FRZB*), rs890711459 (in *FAM160A1*), and rs940913544 (in *HK1*) showed nominal associations with DR risk (*P* = 3.20 × 10^−3^, *P* = 3.58 × 10^−3^, and *P* = 2.76 × 10^−4^, respectively). However, these findings should be interpreted cautiously, as the observed signals do not constitute definitive external replication and provide only limited evidence for consistency.

### Cox Proportional Hazard Analysis for DR

We applied a Cox proportional hazards model to evaluate the associations of *FRZB* and two common SNPs with the longitudinal risk of developing DR. Baseline comparisons showed no significant differences in age at diabetes diagnosis, sex, or HbA1c levels between individuals with and without *FRZB* mutations (Supplementary Table S5). Among 30 individuals carrying *FRZB* mutations, eight developed DR (26.7%), compared with 3312 cases among 33,594 non-carriers (9.9%) (Supplementary Table S6). The analysis indicated that *FRZB* mutations were associated with an increased risk of incident DR (HR = 3.67; 95% CI, 1.83–7.35; *P* = 2.43 × 10^−4^) (Supplementary Table S6). LOVO analyses demonstrated that the association remained significant after excluding any single variant (Supplementary Table S6). Consistently, results from the Firth correction Cox model supported this finding (HR = 3.74; 95% CI, 1.75–6.85; *P* = 0.001) (Supplementary Table S6).

For common variants, 1445 individuals carried the G allele of 4:151662612:C:G in *FAM160A1*, among whom 179 developed DR (12.4%) (Supplementary Table S5). For 10:69300854:A:G in *HK1*, 4731 individuals carried the A allele, with 25 cases of DR (11.0%) (Supplementary Table S5). The Cox analyses showed that both variants were associated with an increased risk of DR: 4:151662612:C:G (HR = 1.32; 95% CI, 1.14–1.54; *P* = 3.31 × 10^−4^); 10:69300854:A:G (HR = 1.19; 95% CI, 1.09–1.31; *P* = 2.45 × 10^−4^).

### Single-Cell Transcriptome Analysis and Virtual Knockout Analysis

**Figure 4.**
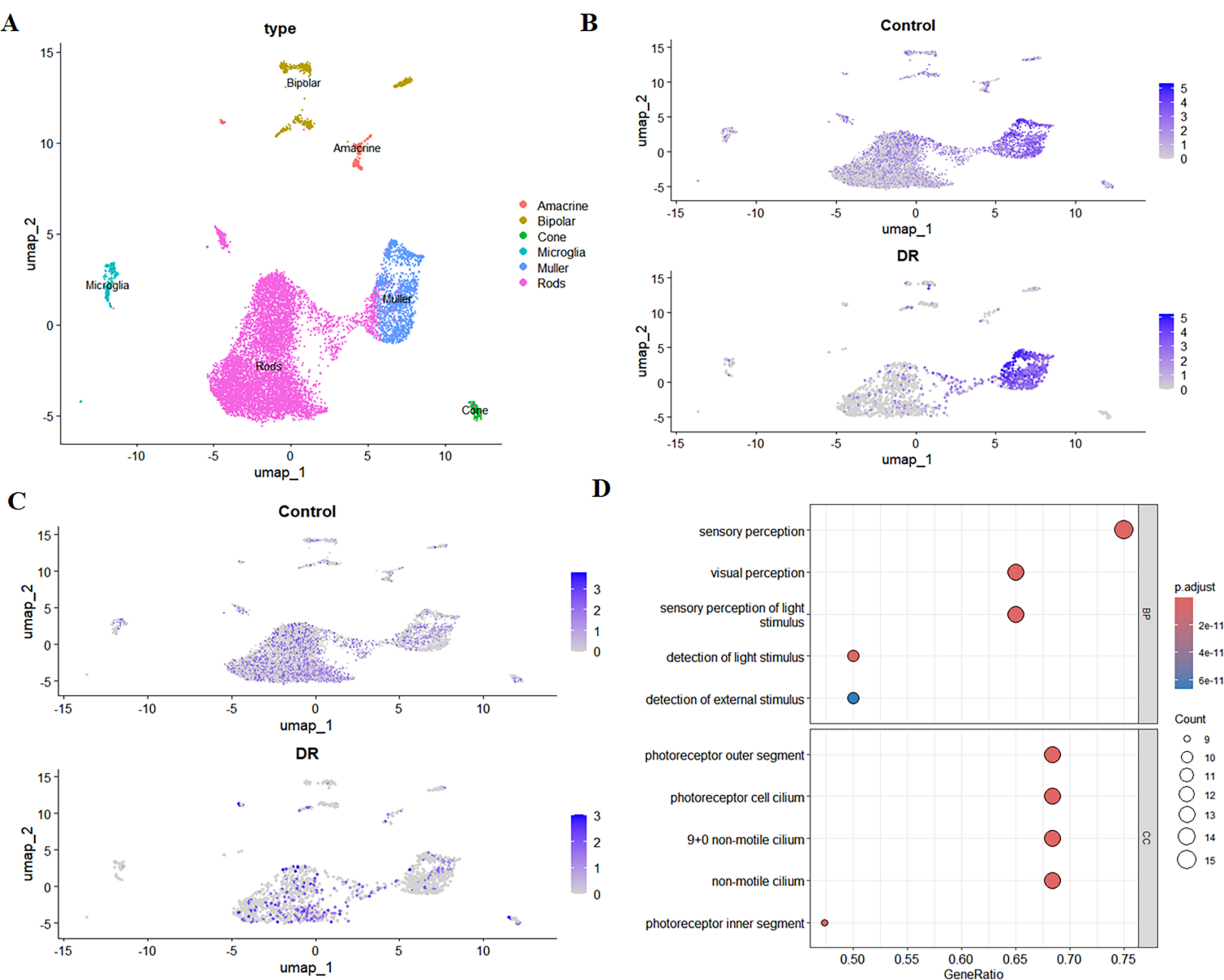
Single-cell transcriptomic profiling and virtual knockout analysis. (**A**) Uniform manifold approximation and projection (UMAP) plot showing the identified cell types. (**B**) UMAP plot of *FRZB* expression in the control and DR groups. (**C**) UMAP plot of *HK1* expression in the control and DR groups. (**D**) Dot plot displaying significantly enriched Gene Ontology (GO) terms based on downstream transcriptional changes identified after the virtual knockout of *FRZB* in bipolar cells.

We annotated six retinal cell types based on marker genes according to the original study, including two glial populations (microglia and Müller glia) and four neuronal cell types (rods, cones, amacrine cells, and bipolar cells) (Fig. 4A). In both bipolar and rod photoreceptors, *FRZB* was significantly downregulated in DR compared to controls, with adjusted *P* values of 4.80 × 10^−6^ (log_2_FC = −0.96) and 2.01 × 10^−30^ (log_2_FC = −0.88), respectively (Fig. 4B). The gene mapped to variant 10:69300854:A:G, *HK1*, was also significantly downregulated in Müller glia (log_2_FC = −1.15, *P*_adj_ = 8.61 × 10^−6^) and rod cells (log_2_FC = −0.60, *P*_adj_ = 7.51 × 10^−6^) (Fig. 4C). In contrast, the gene mapped to variant 4:151662612:C:G, *FAM160A1*, showed no significant changes across cell types.

Following virtual knockout of *FRZB*, a total of 20 differentially affected genes were identified in bipolar cells (Supplementary Table S7). Enrichment analysis indicated that these genes were involved in visual perception, phototransduction, and retinal development (Fig. 4D, Supplementary Table S8). In rod cells, only two differentially affected genes were identified, and no significantly enriched pathways were observed (Supplementary Table S8). For *HK1*, virtual knockout in both rod and Müller cells identified two differentially affected genes in each cell type (Supplementary Table S8), with no significant pathway enrichment detected. Collectively, these findings provide additional functional support for the involvement of *FRZB* in retinal pathology, whereas the roles of *HK1* and *FAM160A1* require further validation.

### Proteomics Analysis and PPI Networks

We evaluated the association between *FRZB* protein levels and the risk of DR in 2647 individuals with type 2 diabetes who had proteomics data available at baseline. During a mean follow-up of 11.94 years, 284 participants developed incident DR. Cox proportional hazards analysis revealed that the higher levels of *FRZB* protein were significantly associated with a reduced risk of DR, with an HR of 0.68 and a 95% CI of 0.48 to 0.97 (*P* = 0.03). We then excluded 13 participants who developed DR within the first year of follow-up and repeated the analysis. The association remained statistically significant, with higher circulating *FRZB* levels associated with a reduced risk of incident DR (HR = 0.67; 95% CI, 0.47–0.97, *P* = 0.03). In the model further adjusted for hypertension status, total cholesterol, and vascular or heart diseases, the association persisted with a similar effect size (HR = 0.67; 95% CI, 0.49–0.99, *P* = 0.047). However, these proteomic findings should be interpreted cautiously, as the observed associations were of modest statistical significance and primarily provide exploratory support for the potential involvement of *FRZB* in DR. Notably, *HK1* and *FAM160A1* were not included in the UK Biobank proteomics dataset and could not be assessed in this analysis.

**Figure 5.**
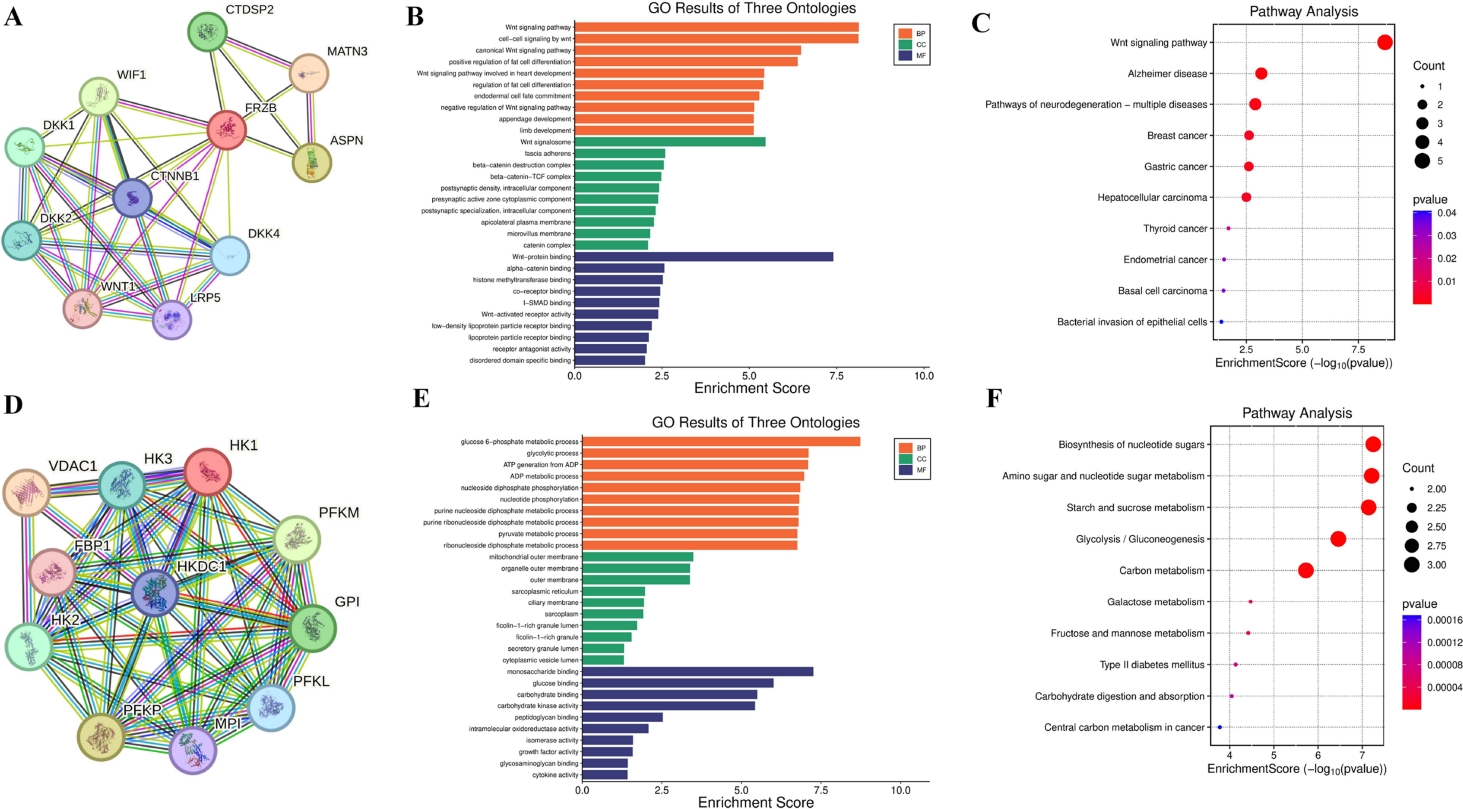
PPI networks and functional pathways of *FRZB* and *HK1*. (**A**) PPI network of *FRZB*. (**B**) DR‑associated genes in the *FRZB* PPI network and GO pathways involving *FRZB*. (**C**) Kyoto Encyclopedia of Genes and Genomes (KEGG) pathways of *FRZB*. (**D**) PPI network of *HK1*. (**E**) DR‑associated genes in the *HK1* PPI network and GO pathways involving *HK1*. (**F**) KEGG pathways of *HK1*.

PPI network analysis was performed for three candidate genes: *FRZB*, *FAM160A1*, and *HK1*. Within the network of *FRZB*, four interacting proteins (CTNNB1, DKK1, LRP5, and WIF1) had prior evidence of association with DR (Supplementary Table S9), pointing to enrichment in the Wnt signaling pathway (Figs. 5A–C). In the network of *HK1*, three interacting proteins (GPI, HK1, and HK2) were supported by existing DR-related evidence (Supplementary Table S9), highlighting pathways related to glycolysis and cellular energy homeostasis (Figs. 5D–F). Notably, *HK1* itself has also been reported to be associated with DR. Both Wnt signaling^32^^–^^34^ and metabolic pathways^35^^,^^36^ have been previously implicated in DR, supporting the biological relevance of these candidate genes. In contrast, no interacting proteins within the PPI network of *FAM160A1* showed prior evidence of association with DR (Supplementary Fig. S2, Supplementary Table S9), indicating that the biological relevance of this candidate requires further validation.

In summary, the gene-based analysis for rare variants suggested that pLoF variants in *FRZB* (MAF < 0.01%) may be associated with an increased risk of DR; however, given the limited external replication evidence, these findings should be interpreted cautiously. The multiomics analyses provided additional exploratory evidence supporting a potential role of *FRZB* in DR. Single-cell transcriptomic and proteomic analyses suggested that higher *FRZB* expression at the RNA and protein levels was associated with a lower risk of DR, which is directionally consistent with the gene-based findings, as loss-of-function variants could reduce *FRZB* activity or expression. In addition, virtual knockout experiments and PPI network analyses yielded convergent exploratory evidence supporting the potential involvement of *FRZB* in DR-related biological processes. We also observed relatively high expression of *FRZB* in retinal tissue (Supplementary Figs. S3, S4). In contrast, the findings for other genes were less consistent across analyses; consequently, these genes were not prioritized as candidate genes in this study.

## Discussion

In this large-scale exome-wide study encompassing approximately 19,000 genes, we employed a dual-control design using both general population controls and diabetes controls to explore genetic associations potentially related to DR. Among the genes identified in the analyses, *FRZB* emerged as a candidate gene of potential interest. Although external replication evidence remained limited, complementary transcriptomic, proteomics, virtual knockout experiments, and PPI network analyses provided exploratory support for a possible role of *FRZB* in DR.

Through gene-based analysis of rare variants, we identified *FRZB* as a candidate gene for DR. *FRZB*, also known as *SFRP3*, is a secreted antagonist of the Wnt signaling pathway, which plays a critical role in ocular vascular development.^37^ Specifically, Wnt signaling regulates key processes in the eye, including regression of the hyaloid vessels vasculature and the formation of organized vascular layers in the retina.^32^^,^^38^ The aberrantly increased Wnt signaling has been implicated in pathological ocular neovascularization, a hallmark of advanced DR.^33^^,^^39^ As an inhibitor of this pathway, reduced expression of *FRZB* may lead to aberrant Wnt activation, potentially contributing to DR pathogenesis. Additionally, system-wide proteomic profiling of the vitreous humor by Alli-Shaik et al.^40^ also identified *FRZB* as being differentially expressed in DR, providing additional circumstantial evidence for its potential involvement in DR, although direct functional studies are needed to confirm this hypothesis.

Through the analysis of common variants, we identified a variant within the *HK1* locus that was associated with an increased risk of DR. *HK1* catalyzes the phosphorylation of glucose to glucose-6-phosphate, representing the initial and rate-limiting step of glycolysis. The genetic findings are consistent with accumulating evidence from previous studies. The integrative GWAS reported that SNPs in metabolic enzyme genes, including *HK1* and *AKR1B1*, may contribute to DR by modulating metabolic pathways such as glucose and cholesterol metabolism, thereby facilitating the systemic transmission of metabolic signals to the retina.^35^ This suggests that *HK1* is not only a key regulator of glucose metabolism but that its genetic variation may also constitute a potential basis for increased susceptibility to DR. Notably, this prior work proposed that genetic variation in *HK1* may reflect a mechanism of DR development that is not solely dependent on hyperglycemia, highlighting a pathway distinct from traditional glucose-driven models.^35^ Consistent with this, our PPI network analysis showed that *HK1* and its interacting partners are enriched in glycolysis and related metabolic processes, in line with its canonical biological role. In addition, previous studies have reported associations between anti-HK1 antibodies and DR.^36^ Together, these findings provide exploratory evidence supporting a role for *HK1* in DR pathogenesis.

We also identified an association between a variant within the *FAM160A1* locus and the risk of DR. However, this finding currently lacks support from multiomics evidence, and no prior studies have reported a link between *FAM160A1* and DR. Further validation and functional characterization are therefore warranted to clarify its potential role in disease pathogenesis.

This study has several notable strengths. First, we conducted a large-scale exome-wide analysis in the UK Biobank, enabling systematic evaluation of coding variation across approximately 19,000 genes in relation to DR risk. In addition, the use of both general population controls and diabetes controls provided complementary comparison frameworks and allowed us to examine the robustness of associations across different control definitions. Second, we incorporated multiple complementary analyses, including transcriptomic, proteomic, virtual knockout, and PPI approaches, to provide exploratory evidence supporting the potential relevance of prioritized genes.

This study also has several limitations, however. First, external validation of the genetic findings remained limited. Although nominally consistent association signals were observed in FinnGen, these findings do not constitute definitive external replication and should be interpreted cautiously. In addition, no experimental validation was performed, and the transcriptomic, proteomic, virtual knockout, and network-based analyses included in this study were exploratory in nature and primarily intended to provide complementary biological context rather than confirm causal mechanisms. Second, the definition of DR in the UK Biobank was based primarily on ICD codes, and standardized ophthalmic grading information distinguishing non-proliferative diabetic retinopathy, proliferative diabetic retinopathy, and diabetes-related macular edema was unavailable. Although we performed additional exploratory analyses using ophthalmic procedure records, these procedures are not specific markers of DR stag. Therefore, the procedure-defined subgroup analyses should not be interpreted as formal disease stratification, and this limitation may affect the interpretation of subtype-related findings. Third, longitudinal assessment of glycemic exposure was limited. HbA1c measurements were largely available only at baseline, and repeat measurements were missing for the majority of participants, limiting our ability to comprehensively evaluate long-term glycemic effects. Finally, the analyses were restricted to individuals of European ancestry because of the limited representation of other populations in the UK Biobank. As a result, the generalizability of these findings to non-European populations remains uncertain. Future studies incorporating diverse ancestries, independent replication cohorts, and functional experimental validation will be important to further clarify the potential role of *FRZB* in DR.

In this large-scale, exome-wide analysis, gene-based analyses identified *FRZB* as a novel candidate gene potentially associated with DR. However, given the limitations in phenotype definition and the lack of definitive external replication, these findings should be interpreted cautiously. Further studies, particularly independent replication and functional experiments, are necessary to clarify the relevance of *FRZB* to DR.

## Supplementary Material

Supplement 1
