# Supplementary material for: Whole-Exome Analysis Identifies Candidate Genes Associated With Diabetic Retinopathy
Source: Invest Ophthalmol Vis Sci. 2026 Jul 8;67(8):25. doi: 10.1167/iovs.67.8.25 (PMC13355387; doi:10.1167/iovs.67.8.25)
Supplement: Supplement 1 [file iovs-67-8-25_s001.docx]

**Table S1**. The definitions of diabetes and diabetic retinopathy.

| Data field | Diabetes | Diabetic retinopathy |
| --- | --- | --- |
| 20002 | 1220, 1222, 1223 | 1275, 1276 |
| 20003 | 1140857494,1140857500,1140868902, | No |
|  | 1140868908,1140883066,1140874646, |  |
|  | 1140874652,1140874658,1140874664, |  |
|  | 1140874674,1140874686,1140874706, |  |
|  | 1140874716,1140874718,1140874744, |  |
|  | 1140874746,1140882964,1140884600, |  |
|  | 1140910564,1140910566,1140910818, |  |
|  | 1140921964,1141152590,1141156984, |  |
|  | 1141157284,1141168660,1141168668, |  |
|  | 1141173882,1141177600,1141177606, |  |
|  | 1141189090,1141189094,1141153254, |  |
|  | 1141171646, 1140800000 |  |
| 41270 | E10-E14 | E103, E113, E123, E133, E143, H360 |
| 41271 | 250 | 2504, 3620 |
| 30740 | >=11.1 mmol/L if fasting time < 8 hours. If fasting time ≥ 8 hours, then ≥7.0 | No |
| 30750 | >= 48 mmol/mol | No |
| 130707  130709  130711  130713  130715 | All | No |

**Table S2**. The OPCS-4 codes for classifying DR subtypes based on ophthalmic interventions.

| Data field | Diabetic retinopathy |
| --- | --- |
| 41272 Operative procedures - OPCS4 | C791 Vitrectomy using anterior approach  C792 Vitrectomy using pars plana approach  C794 Injection into vitreous body NEC  C80 Operations on retinal membrane  C81 Photocoagulation of retina for detachment  C82 Destruction of lesion of retina |

**Table S3**. The summary associations of genes and SNPs with DR and HbA1c Levels.

|  |  |  | **DR vs. DM** | | **DR vs. General** | | **The associations with HbA1c** | |
| --- | --- | --- | --- | --- | --- | --- | --- | --- |
| **Gene/SNP** | **Group** | **Max_maf** | **OR (95% CI)** | **P** | **OR (95% CI)** | **P** | **beta (95% CI)** | **P** |
| FRZB | lof | 0.1% | 1.09 (1.04, 1.15) | 2.55E-07 | 1.13 (1.06, 1.20) | 1.23E-04 | -0.01 (-0.03, 0.01) | 0.63 |
| COX19 | missense;lof | 0.1% | 1.09 (1.02, 1.17) | 0.01 | 1.12 (1.02, 1.22) | 8.45E-08 | 0.01 (-0.02, 0.04) | 0.81 |
| 4:151662612:C:G | - | - | 1.39 (1.17, 1.64) | 1.32E-04 | 1.82 (1.44, 2.30) | 5.83E-07 | 0.02 (-0.05, 0.09) | 0.65 |
| 10:69300854:A:G | - | - | 1.20 (1.09, 1.33) | 2.31E-04 | 1.50 (1.28, 1.75) | 3.35E-07 | -0.53 (-0.57, -0.49) | 3.47E-150 |

**Table S4**. The LOVO results of FRZB.

| **Region** | **Group** | **max_MAF** | **OR (95% CI)** | **Pvalue** |
| --- | --- | --- | --- | --- |
| FRZB_2:182837946:A:C | lof | 0.001 | 1.08 (1.03-1.14) | 7.84E-06 |
| FRZB_2:182838429:A:T | lof | 0.001 | 1.06 (1.00-1.12) | 3.30E-03 |
| FRZB_2:182838468:AG:A | lof | 0.001 | 1.08 (1.03-1.14) | 9.68E-06 |
| FRZB_2:182838525:C:CAA | lof | 0.001 | 1.08 (1.03-1.14) | 9.83E-06 |
| FRZB_2:182838577:C:CACTT | lof | 0.001 | 1.09 (1.03-1.14) | 7.66E-06 |
| FRZB_2:182838615:T:C | lof | 0.001 | 1.09 (1.04-1.15) | 2.78E-07 |
| FRZB_2:182866428:A:AG | lof | 0.001 | 1.25 (1.16-1.34) | 1.81E-09 |

**Table S5**. Baseline characteristics of participants with and without mutations in FRZB and two candidate SNPs.

|  | **Carriers of pLOFs in FRZB** | **Non-carriers of pLOFs in FRZB** | ***P*** |  | **Carriers of the G allele in 4:151662612:C:G** | **Non-carriers of the G allele in 4:151662612:C:G** | ***P*** |  | **Carriers of the A allele in 10:69300854:A:G** | **Non-carriers of the A allele in 10:69300854:A:G** | ***P*** |
| --- | --- | --- | --- | --- | --- | --- | --- | --- | --- | --- | --- |
| n | 30 | 33594 |  |  | 1445 | 32179 |  |  | 4731 | 28893 |  |
| DR, n (%) | 8 (26.7) | 3312 (9.9) | 0.005 |  | 179 (12.4) | 3141 (9.8) | 0.001 |  | 25 (11.0) | 3295 (9.9) | <0.001 |
| Age, years, mean (SD) | 62.40 (9.01) | 59.10 (12.33) | 0.14 |  | 58.95 (12.29) | 59.11 (12.33) | 0.6 |  | 59.15 (12.31) | 59.09 (12.33) | 0.78 |
| Male, n (%) | 17 (56.7) | 20418 (60.8) | 0.78 |  | 867 (60.0) | 19568 (60.8) | 0.56 |  | 2837 (60.0) | 17598 (60.9) | 0.23 |
| HbA1c, mmol/mol, mean (SD) | 44.54 (5.92) | 47.24 (13.25) | 0.26 |  | 47.37 (13.89) | 47.23 (13.22) | 0.70 |  | 47.03 (13.12) | 47.27 (13.27) | 0.26 |

Note: Continuous variables are presented as mean (SD), and categorical variables are presented as n (%).

**Table S6**. Results of Cox proportional hazards model analysis, LOVO analysis, and Firth Cox analysis for mutations (MAF < 0.1% and pLOF) in FRZB and DR.

|  | DR in mutation carriers, n/N (%) | DR in mutation non-carriers, n/N (%) | HR (95% CI) | *P* |
| --- | --- | --- | --- | --- |
| FRZB mutations  (MAF<0.1% and pLOF, Cox) | 8/30 (26.67%) | 3312/33594 (9.86%) | 3.67 (1.83-7.35) | 2.43×10^-4^ |
| Excluding 2:182837946:A:C | 7/29 (24.14%) | 3313/33595 (9.86%) | 3.26 (1.55-6.84) | 1.82×10^-3^ |
| Excluding 2:182838429:A:T | 5/27 (18.52%) | 3315/33597 (9.87%) | 2.48 (1.03-5.97) | 0.04 |
| Excluding 2:182838468:AG:A | 7/29 (24.14%) | 3313/33595 (9.86%) | 3.26 (1.55-6.84) | 1.81×10^-3^ |
| Excluding 2:182838525:C:CAA | 7/29 (24.14%) | 3313/33595 (9.86%) | 3.27 (1.56-6.86) | 1.77×10^-3^ |
| Excluding 2:182838577:C:CACTT | 7/29 (24.14%) | 3313/33595 (9.86%) | 3.27 (1.56-6.87) | 1.74×10^-3^ |
| Excluding 2:182838615:T:C | 8/29 (27.59%) | 3312/33595 (9.86%) | 3.70 (1.85-7.41) | 2.20×10^-4^ |
| Excluding 2:182866428:A:AG | 7/8 (87.50%) | 3313/33596 (9.86%) | 20.98 (9.97-44.11) | 1.02×10^-15^ |
| FRZB mutations  (MAF<0.1% and pLOF, Firth Cox) | 7/29 (24.14%) | 3313/33595 (9.86%) | 3.74 (1.75-6.85) | 1.62×10^-3^ |

**Table S7**. Differentially expressed genes after virtual knockout of candidate genes.

| gene | distance | Z | FC | p.value | p.adj |
| --- | --- | --- | --- | --- | --- |
| **Virtual knockout of FRZB in bipolar cells** | | | | | |
| RP1 | 3.28E-05 | 1.50 | 151.88 | 6.73E-35 | 6.73E-32 |
| CPLX4 | 2.84E-05 | 1.48 | 114.40 | 1.07E-26 | 7.12E-24 |
| ROM1 | 2.80E-05 | 1.47 | 110.89 | 6.27E-26 | 3.13E-23 |
| GNAT1 | 2.75E-05 | 1.47 | 106.85 | 4.79E-25 | 1.92E-22 |
| SAG | 2.60E-05 | 1.46 | 95.64 | 1.38E-22 | 4.60E-20 |
| PRPH2 | 2.23E-05 | 1.44 | 70.21 | 5.34E-17 | 1.53E-14 |
| AIPL1 | 1.93E-05 | 1.42 | 52.60 | 4.09E-13 | 1.02E-10 |
| RCVRN | 1.88E-05 | 1.42 | 50.02 | 1.53E-12 | 3.39E-10 |
| NRL | 1.80E-05 | 1.41 | 45.70 | 1.38E-11 | 2.76E-09 |
| GNGT1 | 1.54E-05 | 1.39 | 33.64 | 6.62E-09 | 1.20E-06 |
| PODXL | 1.47E-05 | 1.38 | 30.65 | 3.09E-08 | 5.16E-06 |
| TULP1 | 1.40E-05 | 1.38 | 27.55 | 1.53E-07 | 2.35E-05 |
| PDC | 1.34E-05 | 1.37 | 25.28 | 4.96E-07 | 7.08E-05 |
| FAIM | 1.28E-05 | 1.37 | 23.02 | 1.60E-06 | 2.14E-04 |
| GRTP1 | 1.25E-05 | 1.36 | 22.24 | 2.41E-06 | 3.01E-04 |
| GNB1 | 1.23E-05 | 1.36 | 21.54 | 3.46E-06 | 4.07E-04 |
| CNGA1 | 1.08E-05 | 1.34 | 16.44 | 5.03E-05 | 5.59E-03 |
| CHD7 | 1.06E-05 | 1.34 | 15.87 | 6.78E-05 | 7.13E-03 |
| PDE6G | 9.88E-06 | 1.33 | 13.83 | 2.00E-04 | 2.00E-02 |
| PDE6A | 9.62E-06 | 1.33 | 13.10 | 2.96E-04 | 2.82E-02 |
| **Virtual knockout of FRZB in rod cells** | | | | | |
| TMSB10 | 2.69E-05 | 3.76 | 1479.17 | 1.48E-323 | 1.48E-320 |
| CWF19L2 | 1.56E-05 | 3.45 | 495.62 | 8.52E-110 | 5.68E-107 |
| **Virtual knockout of HK1 in Müller cells** | | | | | |
| LOC102144419 | 5.22E-06 | 3.13 | 1384.36 | 5.27E-303 | 5.27E-300 |
| RP1L1 | 2.11E-06 | 2.64 | 226.17 | 4.09E-51 | 2.72E-48 |
| **Virtual knockout of HK1 in rod cells** | | | | | |
| CWF19L2 | 1.76E-06 | 3.39 | 1714.11 | 0 | 0 |
| TMSB10 | 5.85E-07 | 2.78 | 190.53 | 2.43E-43 | 1.62E-40 |

**Table S8.** Significant pathways enriched by differentially expressed genes after virtual knockout of FRZB in bipolar cells

|  | ID | Description | GeneRatio | p.adjust | geneID |
| --- | --- | --- | --- | --- | --- |
| BP | GO:0007600 | sensory perception | 15/20 | 1.20E-12 | RP1/CPLX4/ROM1/GNAT1/PRPH2/AIPL1/RCVRN/NRL/TULP1/ |
|  |  |  |  |  | PDC/GNB1/CNGA1/CHD7/PDE6G/PDE6A |
| BP | GO:0007601 | visual perception | 13/20 | 3.21E-12 | RP1/CPLX4/ROM1/GNAT1/PRPH2/AIPL1/RCVRN/NRL/TULP1/ |
|  |  |  |  |  | PDC/CNGA1/PDE6G/PDE6A |
| BP | GO:0050953 | sensory perception of light stimulus | 13/20 | 3.21E-12 | RP1/CPLX4/ROM1/GNAT1/PRPH2/AIPL1/RCVRN/NRL/TULP1/PDC/ |
|  |  |  |  |  | CNGA1/PDE6G/PDE6A |
| BP | GO:0009583 | detection of light stimulus | 10/20 | 3.21E-12 | RP1/ROM1/GNAT1/SAG/PRPH2/AIPL1/RCVRN/GNGT1/TULP1/PDC |
| BP | GO:0009581 | detection of external stimulus | 10/20 | 6.70E-11 | RP1/ROM1/GNAT1/SAG/PRPH2/AIPL1/RCVRN/GNGT1/TULP1/PDC |
| BP | GO:0009582 | detection of abiotic stimulus | 10/20 | 6.70E-11 | RP1/ROM1/GNAT1/SAG/PRPH2/AIPL1/RCVRN/GNGT1/TULP1/PDC |
| BP | GO:0051606 | detection of stimulus | 10/20 | 7.17E-10 | RP1/ROM1/GNAT1/SAG/PRPH2/AIPL1/RCVRN/GNGT1/TULP1/PDC |
| BP | GO:0050877 | nervous system process | 15/20 | 9.48E-10 | RP1/CPLX4/ROM1/GNAT1/PRPH2/AIPL1/RCVRN/NRL/TULP1/PDC/ |
|  |  |  |  |  | GNB1/CNGA1/CHD7/PDE6G/PDE6A |
| BP | GO:0060041 | retina development in camera-type eye | 9/20 | 1.25E-08 | RP1/ROM1/GNAT1/PRPH2/NRL/TULP1/GNB1/CHD7/PDE6A |
| BP | GO:0009584 | detection of visible light | 7/20 | 1.25E-08 | RP1/ROM1/GNAT1/SAG/PRPH2/AIPL1/TULP1 |
| BP | GO:0042461 | photoreceptor cell development | 7/20 | 1.25E-08 | RP1/ROM1/GNAT1/PRPH2/NRL/GNGT1/TULP1 |
| BP | GO:0007602 | phototransduction | 7/20 | 2.89E-08 | RP1/GNAT1/SAG/AIPL1/RCVRN/GNGT1/PDC |
| BP | GO:0046530 | photoreceptor cell differentiation | 7/20 | 2.89E-08 | RP1/ROM1/GNAT1/PRPH2/NRL/GNGT1/TULP1 |
| BP | GO:0009416 | response to light stimulus | 10/20 | 3.33E-08 | RP1/ROM1/GNAT1/SAG/PRPH2/AIPL1/RCVRN/GNGT1/TULP1/PDC |
| BP | GO:0009314 | response to radiation | 10/20 | 1.49E-07 | RP1/ROM1/GNAT1/SAG/PRPH2/AIPL1/RCVRN/GNGT1/TULP1/PDC |
| BP | GO:0001654 | eye development | 10/20 | 2.30E-07 | RP1/ROM1/GNAT1/PRPH2/NRL/GNGT1/TULP1/GNB1/CHD7/PDE6A |
| BP | GO:0150063 | visual system development | 10/20 | 2.30E-07 | RP1/ROM1/GNAT1/PRPH2/NRL/GNGT1/TULP1/GNB1/CHD7/PDE6A |
| BP | GO:0003008 | system process | 15/20 | 2.36E-07 | RP1/CPLX4/ROM1/GNAT1/PRPH2/AIPL1/RCVRN/NRL/TULP1/PDC/ |
|  |  |  |  |  | GNB1/CNGA1/CHD7/PDE6G/PDE6A |
| BP | GO:0048880 | sensory system development | 10/20 | 2.69E-07 | RP1/ROM1/GNAT1/PRPH2/NRL/GNGT1/TULP1/GNB1/CHD7/PDE6A |
| BP | GO:0001754 | eye photoreceptor cell differentiation | 6/20 | 3.11E-07 | RP1/ROM1/GNAT1/NRL/GNGT1/TULP1 |
| BP | GO:0043010 | camera-type eye development | 9/20 | 1.29E-06 | RP1/ROM1/GNAT1/PRPH2/NRL/TULP1/GNB1/CHD7/PDE6A |
| BP | GO:0007423 | sensory organ development | 10/20 | 2.59E-06 | RP1/ROM1/GNAT1/PRPH2/NRL/GNGT1/TULP1/GNB1/CHD7/PDE6A |
| BP | GO:0042462 | eye photoreceptor cell development | 5/20 | 4.23E-06 | RP1/GNAT1/NRL/GNGT1/TULP1 |
| BP | GO:0090596 | sensory organ morphogenesis | 7/20 | 1.53E-05 | RP1/ROM1/GNAT1/NRL/GNGT1/TULP1/CHD7 |
| BP | GO:0048592 | eye morphogenesis | 6/20 | 6.84E-05 | RP1/ROM1/GNAT1/NRL/GNGT1/TULP1 |
| BP | GO:0009628 | response to abiotic stimulus | 10/20 | 7.73E-05 | RP1/ROM1/GNAT1/SAG/PRPH2/AIPL1/RCVRN/GNGT1/TULP1/PDC |
| BP | GO:0060219 | camera-type eye photoreceptor cell differentiation | 4/20 | 8.78E-05 | RP1/ROM1/GNAT1/NRL |
| BP | GO:0008277 | regulation of G protein-coupled receptor signaling pathway | 5/20 | 2.55E-04 | GNAT1/SAG/AIPL1/PDC/PDE6G |
| BP | GO:0007186 | G protein-coupled receptor signaling pathway | 7/20 | 5.12E-04 | GNAT1/SAG/AIPL1/GNGT1/PDC/GNB1/PDE6G |
| BP | GO:0050906 | detection of stimulus involved in sensory perception | 4/20 | 6.49E-04 | ROM1/GNAT1/PRPH2/TULP1 |
| BP | GO:0071482 | cellular response to light stimulus | 4/20 | 8.14E-04 | RP1/GNAT1/SAG/AIPL1 |
| BP | GO:0060042 | retina morphogenesis in camera-type eye | 4/20 | 1.01E-03 | RP1/ROM1/GNAT1/NRL |
| BP | GO:0009605 | response to external stimulus | 11/20 | 2.62E-03 | RP1/ROM1/GNAT1/SAG/PRPH2/AIPL1/RCVRN/GNGT1/TULP1/PDC/CHD7 |
| BP | GO:0003407 | neural retina development | 4/20 | 2.62E-03 | RP1/ROM1/GNAT1/NRL |
| BP | GO:0071478 | cellular response to radiation | 4/20 | 3.03E-03 | RP1/GNAT1/SAG/AIPL1 |
| BP | GO:0048593 | camera-type eye morphogenesis | 4/20 | 4.07E-03 | RP1/ROM1/GNAT1/NRL |
| BP | GO:0009887 | animal organ morphogenesis | 7/20 | 7.12E-03 | RP1/ROM1/GNAT1/NRL/GNGT1/TULP1/CHD7 |
| BP | GO:0071214 | cellular response to abiotic stimulus | 4/20 | 1.84E-02 | RP1/GNAT1/SAG/AIPL1 |
| BP | GO:0104004 | cellular response to environmental stimulus | 4/20 | 1.84E-02 | RP1/GNAT1/SAG/AIPL1 |
| BP | GO:0022008 | neurogenesis | 9/20 | 2.43E-02 | RP1/ROM1/GNAT1/PRPH2/NRL/GNGT1/TULP1/FAIM/CHD7 |
| BP | GO:0001895 | retina homeostasis | 3/20 | 2.45E-02 | RP1/AIPL1/TULP1 |
| BP | GO:0048513 | animal organ development | 11/20 | 2.48E-02 | RP1/ROM1/GNAT1/PRPH2/NRL/GNGT1/PODXL/TULP1/GNB1/CHD7/PDE6A |
| BP | GO:0048468 | cell development | 10/20 | 4.56E-02 | RP1/ROM1/GNAT1/PRPH2/NRL/GNGT1/PODXL/TULP1/FAIM/CHD7 |
| CC | GO:0001750 | photoreceptor outer segment | 13/19 | 1.44E-17 | RP1/ROM1/GNAT1/SAG/PRPH2/RCVRN/GNGT1/TULP1/PDC/ |
|  |  |  |  |  | GNB1/CNGA1/PDE6G/PDE6A |
| CC | GO:0097733 | photoreceptor cell cilium | 13/19 | 3.20E-17 | RP1/ROM1/GNAT1/SAG/PRPH2/RCVRN/GNGT1/TULP1/PDC/ |
|  |  |  |  |  | GNB1/CNGA1/PDE6G/PDE6A |
| CC | GO:0097731 | 9+0 non-motile cilium | 13/19 | 3.38E-17 | RP1/ROM1/GNAT1/SAG/PRPH2/RCVRN/GNGT1/TULP1/PDC/ |
|  |  |  |  |  | GNB1/CNGA1/PDE6G/PDE6A |
| CC | GO:0097730 | non-motile cilium | 13/19 | 9.19E-17 | RP1/ROM1/GNAT1/SAG/PRPH2/RCVRN/GNGT1/TULP1/PDC/ |
|  |  |  |  |  | GNB1/CNGA1/PDE6G/PDE6A |
| CC | GO:0001917 | photoreceptor inner segment | 9/19 | 2.41E-12 | RP1/GNAT1/SAG/PRPH2/AIPL1/RCVRN/GNGT1/TULP1/PDC |
| CC | GO:0005929 | cilium | 13/19 | 1.31E-11 | RP1/ROM1/GNAT1/SAG/PRPH2/RCVRN/GNGT1/TULP1/PDC/ |
|  |  |  |  |  | GNB1/CNGA1/PDE6G/PDE6A |
| CC | GO:0043005 | neuron projection | 14/19 | 1.78E-08 | RP1/CPLX4/ROM1/GNAT1/SAG/PRPH2/RCVRN/GNGT1/TULP1/ |
|  |  |  |  |  | PDC/GNB1/CNGA1/PDE6G/PDE6A |
| CC | GO:0120025 | plasma membrane bounded cell projection | 15/19 | 1.44E-07 | RP1/CPLX4/ROM1/GNAT1/SAG/PRPH2/RCVRN/GNGT1/PODXL/ |
|  |  |  |  |  | TULP1/PDC/GNB1/CNGA1/PDE6G/PDE6A |
| CC | GO:0042995 | cell projection | 15/19 | 2.24E-07 | RP1/CPLX4/ROM1/GNAT1/SAG/PRPH2/RCVRN/GNGT1/PODXL/ |
|  |  |  |  |  | TULP1/PDC/GNB1/CNGA1/PDE6G/PDE6A |
| CC | GO:0042622 | photoreceptor outer segment membrane | 5/19 | 6.69E-07 | ROM1/GNAT1/CNGA1/PDE6G/PDE6A |
| CC | GO:0097381 | photoreceptor disc membrane | 5/19 | 6.69E-07 | GNAT1/GNGT1/GNB1/PDE6G/PDE6A |
| CC | GO:0060170 | ciliary membrane | 5/19 | 5.57E-06 | ROM1/GNAT1/CNGA1/PDE6G/PDE6A |
| CC | GO:0031253 | cell projection membrane | 6/19 | 9.38E-05 | ROM1/GNAT1/PODXL/CNGA1/PDE6G/PDE6A |
| CC | GO:0019897 | extrinsic component of plasma membrane | 3/19 | 2.33E-03 | GNAT1/GNGT1/GNB1 |
| CC | GO:0009898 | cytoplasmic side of plasma membrane | 3/19 | 7.70E-03 | GNAT1/GNGT1/GNB1 |
| CC | GO:0098562 | cytoplasmic side of membrane | 3/19 | 8.51E-03 | GNAT1/GNGT1/GNB1 |
| CC | GO:0019898 | extrinsic component of membrane | 3/19 | 1.24E-02 | GNAT1/GNGT1/GNB1 |
| CC | GO:0098590 | plasma membrane region | 6/19 | 4.59E-02 | ROM1/GNAT1/PODXL/CNGA1/PDE6G/PDE6A |
| CC | GO:0035869 | ciliary transition zone | 2/19 | 4.59E-02 | RP1/GNAT1 |

**Table S9**. The associations between proteins in the PPI network of FRZB, HK1, and FAM160A1 and DR.

|  | **Open targets** | **DIGENET** |  |  | **Open targets** | **DIGENET** |  |  | **Open targets** | **DIGENET** |
| --- | --- | --- | --- | --- | --- | --- | --- | --- | --- | --- |
| **Candidate target: FRZB** | | |  | **Candidate target: HK1** | | |  | **Candidate target: FAM160A1** | | |
| ASPN |  |  |  | FBP1 |  |  |  | AKTIP |  |  |
| CTDSP2 |  |  |  | GPI | √ |  |  | AP4E1 |  |  |
| CTNNB1 |  | √ |  | HK1 | √ |  |  | FAM160A1 |  |  |
| DKK1 | √ | √ |  | HK2 | √ | √ |  | FAM160B1 |  |  |
| DKK2 |  |  |  | HK3 |  |  |  | GULP1 |  |  |
| DKK4 |  |  |  | HKDC1 |  |  |  | HOOK1 |  |  |
| LRP5 | √ | √ |  | MPI |  |  |  | HOOK2 |  |  |
| WIF1 | √ | √ |  | PFKL |  |  |  | LZTS2 |  |  |
| MATN3 |  |  |  | PFKM |  |  |  | HOOK3 |  |  |
| WNT1 |  |  |  | PFKP |  |  |  | GPR26 |  |  |
| FRZB |  |  |  | FBP1 |  |  |  | HOOK3 |  |  |

√ indicates genes associated with DR found in Open Targets or DIGENET.


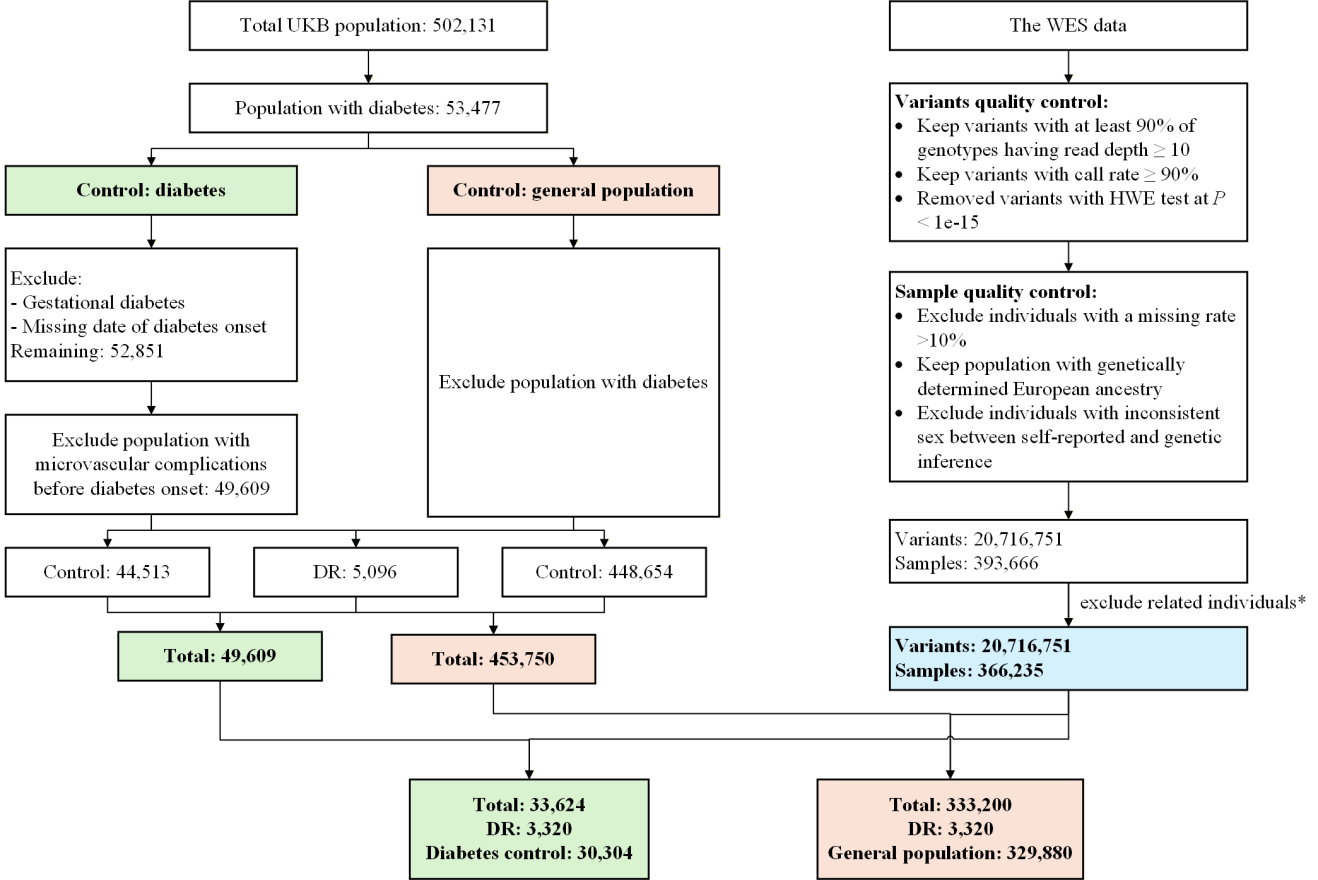


**Figure S1**. The study population and quality control of the whole exome sequencing data.

DR: diabetic retinopathy; WES: whole exome sequencing; HWE: Hardy-Weinberg equilibrium. *The related individuals were defined as a KING kinship coefficient ≥ 0.0884, equal to second-degree relatedness or closer.


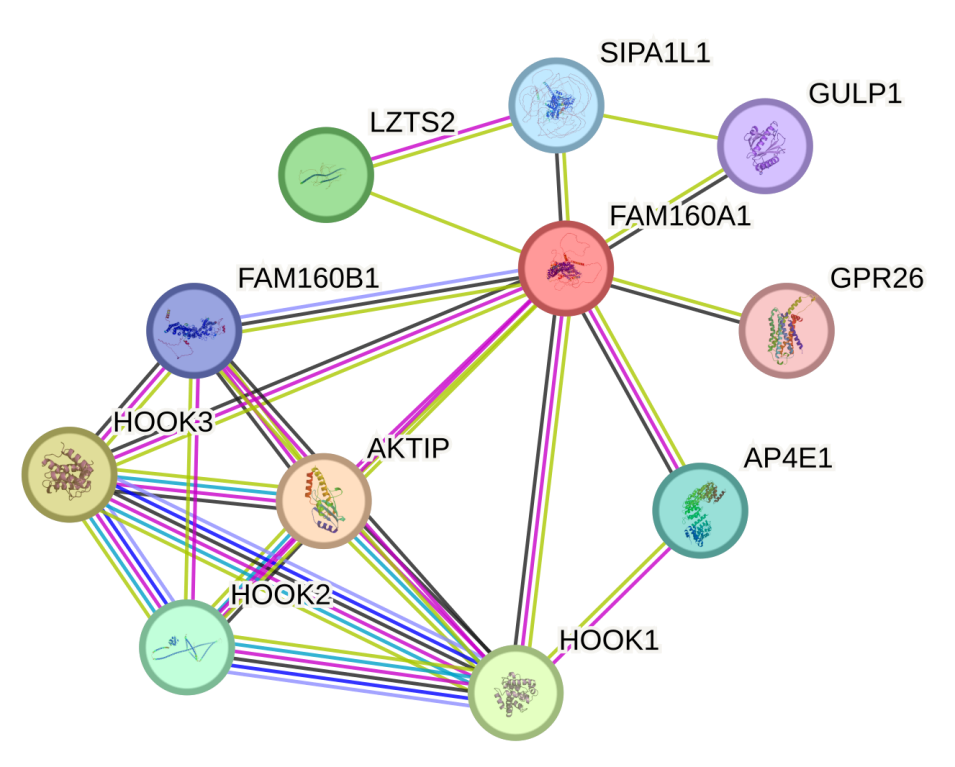


**Figure S2**. The PPI networks of the gene FAM160A1.


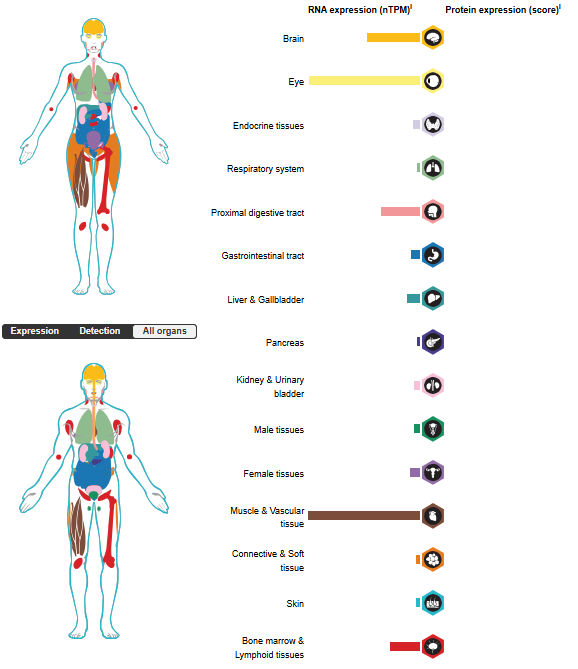


**Figure S3.** FRZB RNA expression across all organs. Source: Human Protein Atlas (https://www.proteinatlas.org/).


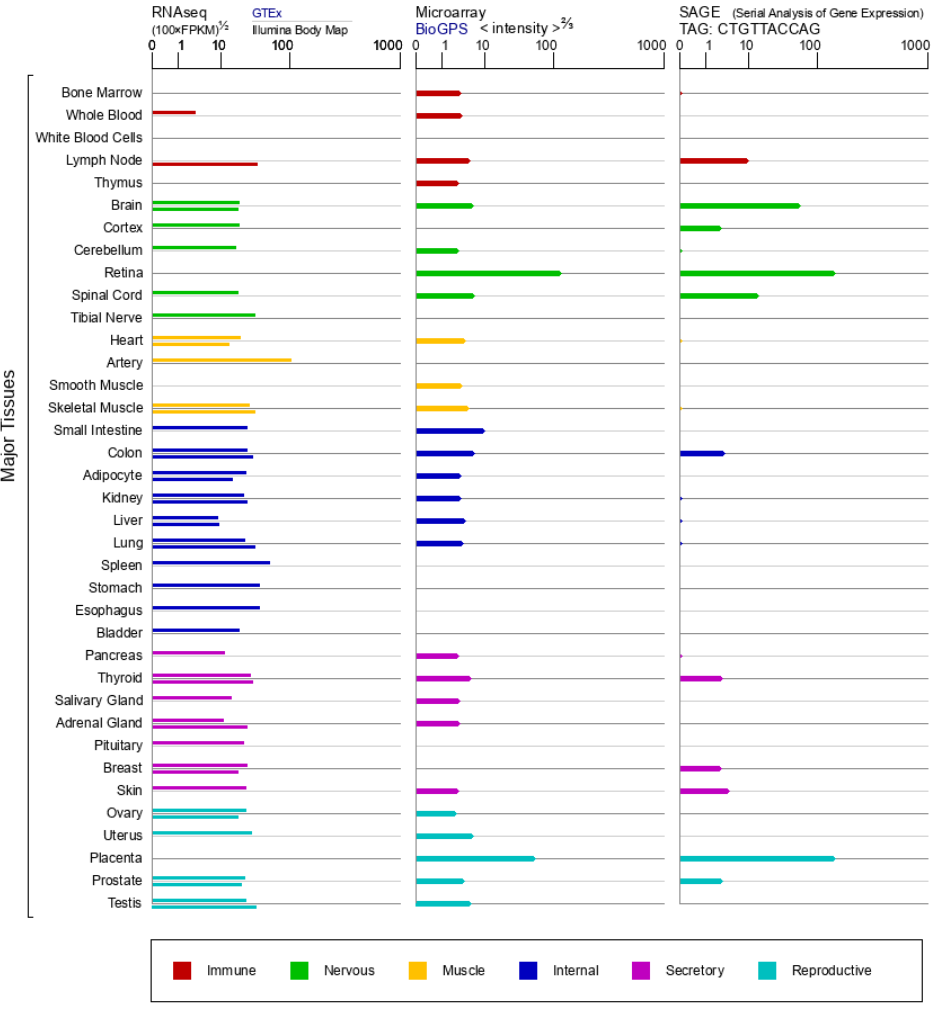


Figure S4. FRZB RNA expression in major tissues.Source: <https://www.genecards.org/.>
